# Hematopoietic Niche Hijacking in Bone Metastases: Roles of Megakaryocytes, Erythroid Lineage Cells, and Perivascular Stromal Subsets

**DOI:** 10.3390/biomedicines14010161

**Published:** 2026-01-12

**Authors:** Abdul Rahman Alkhatib, Youssef Elshimy, Bilal Atassi, Khalid Said Mohammad

**Affiliations:** Department of Anatomy, College of Medicine, Alfaisal University, Riyadh 11533, Saudi Arabia; aralkhatib@alfaisal.edu (A.R.A.); yelshimy@alfaisal.edu (Y.E.); batassi@alfaisal.edu (B.A.)

**Keywords:** bone metastasis, hematopoietic stem-cell niche, megakaryocytes, erythroid-derived myeloid cells, perivascular stroma

## Abstract

Bone metastases mark a critical and often terminal phase in cancer progression, where disseminated tumor cells (DTCs) manage to infiltrate and exploit the complex microenvironments of the bone marrow. While most current therapies focus on the well-known late-stage “vicious cycle” of osteolysis, they often overlook the earlier stages, namely, tumor cell colonization and dormancy. During these early phases, cancer cells co-opt hematopoietic stem cell (HSC) niches, using them as sanctuaries for long-term survival. In this review, we bring together emerging insights that highlight a trio of underappreciated cellular players in this metastatic takeover: megakaryocytes, erythroid lineage cells, and perivascular stromal subsets. Far from being passive bystanders, these cells actively shape the metastatic niche. For instance, megakaryocytes and platelets go beyond their role in transport; they orchestrate immune evasion and dormancy through mechanisms such as transforming growth factor-β1 (TGF-β1) signaling and the physical shielding of tumor cells. In parallel, we uncover a distinct “erythroid-immune” axis: here, stress-induced CD71^+^ erythroid progenitors suppress T-cell responses via arginase-mediated nutrient depletion and checkpoint engagement, forming a potent metabolic barrier against immune attack. Furthermore, leptin receptor–positive (LepR^+^) perivascular stromal cells emerge as key structural players. These stromal subsets not only act as anchoring points for DTCs but also maintain them in protective vascular zones via CXCL12 chemokine gradients. Altogether, these findings reveal that the metastatic bone marrow niche is not static; it is a highly dynamic, multi-lineage ecosystem. By mapping these intricate cellular interactions, we argue for a paradigm shift: targeting these early and cooperative crosstalk, whether through glycoprotein-A repetitions predominant (GARP) blockade, metabolic reprogramming, or other niche-disruptive strategies, could unlock new therapeutic avenues and prevent metastatic relapse at its root.

## 1. Introduction

Bone metastases are a major driver of morbidity and mortality across solid tumors, producing skeletal-related events that include intractable pain, pathologic fracture, spinal cord compression, and hypercalcemia with downstream losses in quality of life and overall survival [1]. Across disease sites, bone is a frequent destination for DTCs: breast and prostate cancers show a pronounced predilection for skeletal colonization, and bone involvement is also common in lung, thyroid, and renal malignancies [2]. This predilection reflects both tumor-intrinsic tropisms and properties of the bone marrow microenvironment that provide a receptive “soil” for DTC survival and growth [3]. Within marrow, endothelial, stromal, and immune compartments form specialized niches that DTCs can exploit to persist, traffic, or reactivate under selective pressures [4].

Historically, the biology of bone metastasis has been framed by the “vicious cycle” model in which tumor-secreted factors stimulate osteoclasts to resorb bone, releasing growth factors that further fuel tumor outgrowth, while osteoblast responses incompletely restore bone integrity [3]. This paradigm underpins approved interventions that target osteoclast activity and RANKL signaling to reduce skeletal-related events in patients with metastatic bone disease [5]. Concurrently, decades of HSC research have defined osteoblastic and vascular/perivascular niches as central organizers of marrow function, shaping how we think about stromal cues that might also condition tumor behavior [6]. Recent spatial and single-cell atlases of normal human and murine marrow extend this view by resolving the cellular geography of niche constituents at high resolution.

Despite this progress, several hematopoietic niche populations with potential relevance to metastasis remain relatively underexplored, specifically megakaryocytes, erythroid-lineage subsets, and defined perivascular stromal ensembles [7,8,9]. Megakaryocytes (MKs) do more than produce platelets: high-ploidy MKs actively regulate hematopoiesis and niche tone, positioning them to influence tumor–niche crosstalk at vascular interfaces [10]. Spatial transcriptomic profiling has begun to map MK programs by maturation state and vascular adjacency, highlighting matrix–cytoskeletal modules that couple MK position to proplatelet formation [11]. Perivascular LepR^+^/CXCL12-abundant stromal cells, which organize HSC retention and differentiation cues, constitute another axis through which tumor cells may access trophic cytokines and chemokine gradients within marrow [12].

Erythroid-lineage cells add an immune dimension: human and murine studies show that CD71^+^ circulating erythroid cells (CECs) can suppress T-cell effector functions via metabolic and checkpoint-mediated mechanisms [13]. Moreover, tumor-conditioned erythroid progenitors can transdifferentiate into erythroid-derived myeloid cells (EDMCs) that express programmed death-ligand 1 (PD-L1) and arginase, thereby curtailing responses to programmed cell death-1 (PD-1)/PD-L1 blockade in preclinical and translational settings [14]. Simultaneously, tumor trafficking to and within bone appears to leverage endothelial ligands and chemokine axes that are integral to marrow physiology, including E-selectin and CXCL12/SDF-1, reinforcing the notion that metastatic cells co-opt native homing circuits [15]. Collectively, these advances suggest that MKs, erythroid subsets, and perivascular stromal cells do not simply coexist with metastases but may actively shape immune contexture, vascular access, and growth-supportive signaling within the skeletal niche [3] (Table 1).

This review seeks to achieve three primary aims: First, we synthesize recent mechanistic work implicating MKs, erythroid programs (including CECs/EDMCs), and LepR^+^/perivascular stromal subsets in tumor–niche interactions that are specific to the bone marrow microenvironment [2,16,17]. Second, we highlight key experimental and translational gaps, particularly the absence of single-cell spatial atlases that co-register tumor, stromal, and erythroid compartments in human bone metastasis tissue [18]. Finally, we propose testable therapeutic entry points that emerge from niche-focused biology, including selective TGF-β activation blockade (e.g., anti-GARP strategies), arginase inhibition, and disruption of marrow trafficking via CXCR4/E-selectin targeting [19,20,21].

## 2. The Bone Marrow Niche: Architecture and Cellular Complexity

### 2.1. Anatomy of the Marrow Niche: Endosteal, Vascular, and Perivascular Compartments

The marrow niche comprises endosteal and vascular/perivascular territories. Each contains distinct stromal and endothelial ensembles that support hematopoiesis [2].

Spatially resolved single-cell maps delineate two major CXCL12-rich perivascular stromal subsets: Osteo-CAR cells, which are enriched toward arteriolar/endosteal regions, and Adipo-CAR cells, which are concentrated around sinusoids. This organization reveals vessel-aligned heterogeneity in both factor production and localization [2]. In humans, a conserved CXCL12-high LEPR^+^ CAR-like stromal population co-expresses SCF and lineage regulators and retains adipogenic/osteogenic potential, substantiating a core perivascular reservoir for HSC maintenance [22]. In in vivo mouse marrow, endothelial and LepR+ stromal compartments act as principal angiocrine sources. While both express high levels of Scf, the LepR+ cells produce the highest concentration of Cxcl12, functionally coupling vascular architecture to HSC retention and support [23]. Functionally, arteriolar versus sinusoidal territories exhibit divergent biases arteriolar domains tending to preserve quiescent HSC pools while sinusoids favor egress and trafficking, an overview consolidated by recent integrative atlases [24].

### 2.2. Classical HSC Regulation vs. Emerging Niche Cell Populations

Emerging evidence indicates that the bone marrow endothelium forms anatomically distinct vascular niches that spatially orchestrate HSC quiescence, retention, and lineage output. In situ imaging atlases in mice confirm this diversification by showing lineage programs that map to specific vessel types (e.g., M-CSF^+^ vascular segments supporting monocyte/dendritic trajectories), emphasizing how endothelial geography organizes hematopoiesis [25]. Beyond SCF, endothelial ANGPTL2 sustains HSC function, emphasizing a broader pro-hematopoietic secretome from the vascular wall [23]. Megakaryocytes co-localize with sinusoids and restrain the HSC pool in mice [19,26,27]. MKs act as negative regulators of HSC function [19]. Together, this architecture defines vessel-type–specific microenvironments that gate HSC quiescence, retention, and egress.

### 2.3. Concept of Niche Hijacking in Metastasis

LepR^+^ stroma and CAR cells form a core HSC retention hub [23]. Single-cell mapping resolves Osteo-CAR (arteriolar) and Adipo-CAR (sinusoidal) niches [2]. In human marrow, a conserved CXCL12-high LepR^+^ CAR-like population co-expresses SCF and lineage regulators and retains adipo-osteogenic potential, confirming a shared perivascular stromal archetype [22]. Lineage tracing plus single-cell RNA-seq in mice further reveal that LepR^+^ stroma is heterogeneous, comprising skeletal/stromal progenitors that bias toward osteogenic or adipogenic programs under homeostasis or stress [28]. In mice, the endothelium supplies membrane-bound SCF early postnatally, while LepR^+^ cells provide the highest CXCL12 [23]. Perivascular adiponectin-lineage progenitors are a major M-CSF source in vivo in mice [29]. This links stromal composition to monocyte/osteoclast support. In single-cell studies of human bone metastases, the stromal compartment appears to undergo a characteristic remodeling [30]. DTCs are closely linked to the emergence of a tumor-associated mesenchymal stromal cell (TA-MSC) subset that shows features of cancer-associated fibroblasts, promotes epithelial–mesenchymal transition (EMT), and disrupts RANK/RANKL/OPG signaling in a way that favors osteoclastic bone resorption [30]. These TA-MSC programs replace normal LepR^+^/CAR-cell hematopoietic support functions with aberrant cytokine loops that enhance tumor survival and suppress immune surveillance [22,30]. Functionally, tumor cells use E-selectin for marrow entry and CXCL12–CXCR4 for retention. In mouse prostate and breast cancer models, CXCR4 or dual E-selectin/CXCR4 blockade reduces intraosseous colonization or mobilizes established disease; pharmacologic CXCR4 blockade (plerixafor/balixafortide) or dual E-selectin/CXCR4 antagonism reduces intraosseous colonization and/or mobilizes established marrow disease [31,32,33].

## 3. Megakaryocytes in Bone Metastases

### 3.1. Physiological Roles: Megakaryocytes as Platelet Factories and Niche Modulators

MKs physically interact with HSCs in mouse bone marrow to maintain quiescence; consequently, genetic deletion of MKs causes HSC activation and proliferation. According to RNA-sequencing data, MKs express Tgfb1 at substantially higher levels than many other stromal niche cells. When MKs are ablated in mice, biologically active TGF-β1 protein in bone marrow drops, and nuclear localization of phosphorylated SMAD2/3 (pSMAD2/3) in HSCs is reduced, indicating MK-derived TGF-β1 mediates HSC quiescence via SMAD signaling in mice [34,35]. Beyond TGF-β1, murine MKs classically enforce quiescence via platelet factor-4 (PF4/CXCL4); disruption of this axis enhances HSC cycling, creating a physiological standard for MK-mediated niche regulation [26,27]. While these mechanisms are primarily defined in murine models, the spatial proximity between MKs and vWF + HSCs has been histologically confirmed in human bone marrow, suggesting that the MK–niche axis is a conserved feature of human hematopoiesis [36]. This homeostatic output is context-dependent: in response to stress (irradiation or chemotherapy), MKs shift from secreting quiescence factors to releasing FGF1 and IGF1 to promote HSC regeneration in mice [10]. High-ploidy, large-cytoplasmic megakaryocytes (LCMs) are critical negative regulators of HSC function, the absence of which results in a pronounced increase in bone marrow HSCs concurrent with endogenous mobilization in mice [19]. Structurally, Megakaryocytes are physically ensheathed within a perivascular extracellular-matrix “cage” that stabilizes their location at vascular interfaces and facilitates local delivery of MK-secreted factors to adjacent cells [37]. These regulatory functions are often subverted in malignancy; for example, loss of Erbin in megakaryocytes/platelets suppresses colorectal cancer metastasis by enhancing B-cell–mediated antitumor immunity in mice [38]. Studies isolating cells from metastatic patient blood samples report increased occurrence of circulating megakaryocytes and megakaryocyte–tumor cell associations, confirming the idea that during dissemination, MKs (or MK fragments/platelets) physically engage with tumor cells [39].

### 3.2. MK-Derived TGF-β: Dormancy Versus Immune Suppression

Megakaryocyte and platelet-derived TGF-β1 acts as a negative regulator, inhibiting the production of new megakaryocytes in the bone marrow and increasing thrombopoietin (TPO) expression in the liver [40]. In mouse bone metastasis models, DTCs exploit osteoclast- and platelet-derived TGF-β to maintain quiescence and drug tolerance [41]. In addition, Platelet-derived TGF-β1 is a potent immunosuppressive factor that enhances myeloid-derived suppressor cell function in preclinical models via canonical TGF-β/Smad signaling [42]. Megakaryocytes and platelets store latent TGF-β1 in α-granules and release active TGF-β1 upon activation, providing a rapid route for local increases in bioactive ligand in marrow and circulation [42,43,44]. In the advanced stage of cancer, platelet-derived TGF-β acts in a pro-tumorigenic and pro-metastatic manner, promoting malignant phenotypes such as Epithelial-to-Mesenchymal Transition (EMT), cancer stemness, and angiogenesis [42,45,46]. In preclinical studies, pharmacologic suppression of TGF-β1 signaling was demonstrated to reverse the platelet-induced functional reprogramming of myeloid-derived suppressor cells (MDSCs), thereby confirming the strong immunosuppressive effect of the MK/platelet TGF-β axis [42]. Together, these results demonstrate that, depending on the temporal and spatial dynamics of its release, TGF-β1 generated via MK and platelets can either induce dormancy or establish a permissive immunosuppressive habitat. Selective inhibition of platelet-activated TGF-β1 is feasible: blocking the latent TGF-β1 docking receptor GARP limits TGF-β1 activation without broad cytopenias and improves antitumor immunity in mouse models [47]. Additionally, in certain models, intratumoral T-cell infiltration is increased when GARP: TGF-β1 blocking and PD-1 inhibition are combined, indicating a molecular connection between Treg-derived TGF-β1 and checkpoint resistance [48]. Because GARP concentrates latent TGF-β1 on Tregs (and also MKs/platelets), its blockade dampens local TGF-βactivation without globally suppressing hematopoiesis in murine PMF [47]. Mechanistically, diet-induced preactivation of platelets and endothelium created a permissive premetastatic niche in vivo, and a short anti-platelet antibody pulse markedly reduced tumor-cell homing and early metastatic outgrowth in the liver [49]. Translationally, a first-in-human trial of the anti-GARP:TGF-β1 antibody livmoniplimab rapidly saturated circulating platelet GARP–TGF-β1 complexes and produced objective responses in combination with anti-PD-1, supporting selective interruption of platelet-activated TGF-β1 signaling [50].

### 3.3. Platelet–Megakaryocyte–Tumor Axis and Thrombopoiesis

The tumor microenvironment establishes a cooperative megakaryocyte–platelet axis that critically promotes metastasis. Clinical studies of metastatic prostate cancer demonstrate that circulating tumor cells (CTCs) are frequently cloaked by platelets, a phenomenon that shields them from immune surveillance [51,52,53]. Tumor-induced platelet activation facilitates extravasation: activated platelets provide adhesive and proteolytic functions that enable tumor cell arrest, transendothelial migration, and seeding in target tissues [54,55]. Quantitative studies in men with metastatic prostate cancer show a positive correlation between CTC numbers and absolute platelet counts, supporting a clinical link between thrombopoiesis and CTC burden [56]. These results highlight the platelet–megakaryocyte–tumor axis as a key modulator of metastatic spread. Tumors actively bias hematopoiesis toward megakaryopoiesis. Tumor-released kynurenine engages the aryl hydrocarbon receptor (AhR) in megakaryocyte–erythroid progenitors (MEPs), reprogramming RUNX1 activity and skewing differentiation toward megakaryocytes, thereby expanding the platelet pool available to assist dissemination [57]. Single-cell lineage capture in mice further shows fate-specific regulatory rewiring across hematopoietic trajectories, supporting context-dependent shifts into the megakaryocytic lineage under tumor-derived cues [57]. At the level of mature LCMs are key platelet factories and niche regulators [19], while their perivascular ECM “cage” architecture may anchor MKs at vascular interfaces where proplatelet shedding is optimized [37]. Collectively, these data outline direct tumor/progenitor (MEP) and tumor/MK circuitries that elevate platelet biogenesis. Beyond local marrow skewing, systemic cytokine loops drive paraneoplastic thrombocytosis, a poor-prognosis phenotype across multiple solid tumors. Tumor- and stromal-derived IL-6 can induce hepatic thrombopoietin (TPO), amplifying platelet production; in parallel, tumor-secreted metabolites (e.g., kynurenine) bias MEP fate as above, providing complementary marrow-intrinsic reinforcement [57]. Clinically, elevated platelets track with higher CTC numbers and worse outcomes [53,56], consistent with a feed-forward thrombopoietic program that fuels metastatic competence.

In the bloodstream, platelets rapidly form physical aggregates (microthrombi) around CTCs, shielding them from shear stress and NK-cell cytotoxicity [46,49]. This aggregate formation is mediated by P-selectin (CD62P) on activated platelets, binding PSGL-1 on tumor cells to initiate tethering and rolling; increased P-selectin ligand density on cancer cells augments adhesive capture under flow [58]. Stable adhesion and vascular arrest are then reinforced by integrin αIIbβ3 (GPIIb/IIIa) on platelets, bridging fibrinogen/fibronectin to tumor and endothelial integrins, and by GPIbα–von Willebrand factor interactions at sites of high shear [58,59]. Platelets also transfer MHC-I and immunomodulatory cargos to tumor cells [44].

Spatial transcriptomics at single-cell resolution in murine marrow reveals megakaryocyte subsets stratified by maturation state and vascular adjacency, with graded expression of Pf4, Itga2b, Vwf, and cytoskeletal/ECM genes aligned to proplatelet formation near sinusoids [60]. Three-dimensional imaging shows MKs encased within a specialized ECM lattice that controls maturation and anchoring to the vascular niche [37], while MKs exert niche-regulatory effects on hematopoiesis [19]. These spatial programs provide anatomical architecture for tumor-driven thrombopoiesis and for efficient platelet handoff to intravascular CTCs at sinusoidal outlets.

### 3.4. Evidence of Tumor-Driven Megakaryopoiesis and MK Heterogeneity

Tumor cells release the tryptophan metabolite kynurenine (Kyn) into the circulation during tumor growth, producing detectable increases in plasma Kyn in tumor-bearing mice and patients [57,61,62]. In MEPs, intracellular Kyn binds and activates the aryl hydrocarbon receptor (AhR), triggering AhR nuclear translocation and target gene induction [57]. The transcriptional program is shifted toward megakaryocytic development in MEPs by activated AhR, which directly increases RUNX1 transcriptional activity. Pharmacologic inhibition of AhR or genetic interference with the AhR–RUNX1 axis reduces tumor-driven megakaryopoiesis and restores normal platelet counts in several tumor-bearing mice models [57]. Specific MK transcriptional states that change according to anatomical location (proximal vs. distal regions) within the bone are revealed by spatial transcriptomic profiling of murine bone marrow megakaryocytes at single-cell resolution [60]. These spatial studies supported the actual positional presence of heterogeneity along the bone’s axis by demonstrating that MKs in the proximal area of the femur express higher levels of pro-platelet genes (such as Ppbp and Pf4), whereas MKs in the distal sections of the femur exhibit distinct functional gene sets, effectively defining distinct pro-platelet and niche-supporting MK subgroups [60]. Researchers found local increases in marrow megakaryocyte numbers in metastatic areas compared to non-metastatic bone in several preclinical models of breast cancer bone metastasis, suggesting MK recruitment or local expansion near DTCs [63]. However, the research lacks conclusive in vivo clonal tracing studies in tumor metastasis models, and there is currently limited clear lineage-tracing evidence that particular MK subgroups directly produce a metastasis-permissive niche (as opposed to growing reactively to tumor presence) [64]. Therefore, identifying the causal role of specific MK subgroups in niche development rather than reactive expansion remains a crucial research problem.

### 3.5. Methodological Challenges in Modeling MK-Niche Interactions

Megakaryocyte genetic ablation in mice results in detectable alterations in HSC behavior, proving that MK disturbance is experimentally manageable [65]. However, these MK lineage-tracing models were not originally intended to examine MK–DTC interactions during cancer metastasis due to their exclusive focus on physiological processes such as HSC differentiation and steady-state thrombopoiesis, indicating a significant research gap [65]. Some hematopoietic lineages exhibit off-target activity in the PF4(CXCL4)-Cre system, which is often used for MK- or platelet-restricted targeting. This highlights the need for additional specialized genetic tools to analyze MK–tumor interactions [66]. New single-cell lineage-tracing (scLT) and genetic barcoding technologies, such as CellTag-multi, exist that can resolve progenitor cell lineage priming in vivo, but their application to complex cell behaviors, such as MK clonal dynamics during hematopoiesis, presents ongoing challenges [67]. Beyond tracing limitations, multiple studies have shown that megakaryocytes suppress osteoclast differentiation and bone resorption in vitro and in vivo [68,69,70,71,72]. Despite these insights, conditional MK perturbation models along with metastatic tracing frameworks will be necessary to define the causative MK–DTC interaction in situ.

### 3.6. Megakaryocytes as Niche “Gatekeepers”

Through signaling pathways, new treatments are being developed to target the detrimental roles of platelets and megakaryocytes in cancer development. Similarly, anti-GARP is used to selectively prevent TGF-β1 activation derived from MK and platelets: TGF-β1 antibodies provide a method to counteract MK-derived immunosuppressive signaling by reviving intratumoral CD8^+^ T cell responses without causing hematological collapse [47]. In addition, preclinical studies targeting the platelet-specific receptor glycoprotein VI (GPVI) with an antibody impaired the outgrowth of established metastases and did so without inducing collateral hemostatic perturbations, suggesting a viable platelet-sparing anti-metastasis approach [73]. Time-limited blocking investigations in mice imply that targeting platelet–tumor interactions at specific times (e.g., perioperative or peri-seeding intervals) may offer antimetastatic benefit while maintaining long-term platelet numbers and function [73].

## 4. Erythroid Lineage Cells and Circulating Erythroid Cells (CECs)

### 4.1. Erythropoiesis and Stress Responses

Erythroblasts mature in perivascular “islands” near CXCL12-rich sinusoidal stroma [74,75,76,77]. Erythroid progenitors occupy distinct perivascular and endosteal niches and exhibit positional transcriptional programs that reflect local oxygenation and stromal signals, according to single-cell and spatial atlases of human and murine bone marrow [2]. The main systemic cause of stress erythropoiesis is erythropoietin (EPO) signaling; elevated levels of EPO in the blood during anemia or hypoxia increase the number of erythroid progenitors and the production of erythroblasts in the bone marrow (and splenic extramedullary locations in mice) [78]. However, this expansion can be impaired by systemic disease states and inflammation, as seen in models of intravascular hemolysis [78].

### 4.2. Emerging Immunological Roles of Immature Erythroid Cells

Immature CECs are an emerging immune-suppressive population that expands in pathological states including cancer and anemia, directly inhibiting T-cell immunity. In anemic and tumor-bearing hosts, immature CECs proliferate and exhibit elevated levels of reactive oxygen species (ROS) and arginase-2 (ARG2), which deplete L-arginine and hinder T-cell proliferation [79]. Compared to more mature erythroid cells, CD71^+^ CECs express PD-L1 (and PD-L2) at higher levels, which allows them to interact with T lymphocytes via PD-1 and inhibit effector function through checkpoint pathways [80]. In clinical and murine models, CD71^+^ erythroid cells suppress T-cell proliferation and IFN-γ via ARG1/2-mediated L-arginine depletion, ROS, and checkpoint engagement [81]. Drivers include EPO-driven stress erythropoiesis, inflammatory cytokines (TNFα/IL-1β), chemotherapy-induced MEP expansion, and tumor-derived GM-CSF that promotes EDMC conversion, observed across preclinical models and patient samples [9,79,82,83]. In mouse models of anemia, expanded CD71+ erythroid cells cause down-regulation of T-cell CD3ζ chain (a marker of T-cell suppression) and impair T-cell proliferation via Arg/ROS-mediated mechanisms [79]. In cancer, tumor-conditioned erythropoiesis and systemic inflammation foster accumulation of CD71^+^ erythroid populations that are detectable in patient blood and in the spleen and bone marrow of tumor-bearing mice [80]. Lineage-tracking in human cancer patients and tumor-bearing mice demonstrates that a sizable fraction of CD45+ erythroid progenitors lose erythroid potential and adopt myeloid transcriptional programs, producing EDMCs with immunosuppressive phenotypes [14]. Depletion or functional blockage of EDMC-like populations in the Cancer Cell study explicitly linked EDMCs to checkpoint resistance by restoring T-cell responses and partially restoring sensitivity to anti-PD-1/PD-L1 therapy in animal models [14]. This is supported by the proliferation of immature CD71^+^ erythroid cells in the spleen and blood of cancer patients and tumor-bearing animals, which suggests tumor-driven extramedullary erythropoiesis [80,84]. This erythroid-to-myeloid transdifferentiation yields PD-L1^+^/arginase^+^ EDMC-like states that blunt PD-1/PD-L1 therapy in vivo and correlate with immune dysfunction in patients [14]. Notably, CEC/EDMC-mediated suppression is reversible in preclinical systems: arginase inhibition or L-arginine repletion restores T-cell proliferation and can synergize with PD-1/PD-L1 blockade; selective anti-CD71 depletion similarly rescues CD8^+^ responses [80,85].

### 4.3. Relevance in Bone Metastasis: How Erythroid Subsets May Dampen Immune Surveillance

Evidence from hematopoiesis:

In marrow, erythropoiesis is organized in perivascular/sinusoidal “islands”, where erythroblasts mature adjacent to venous sinusoids and perivascular stroma; recent single-cell/spatial atlases confirm programs concentrated in sinusoid-proximal territories, particularly in adult/aging human marrow [2]. CECs—an immature CD71^+^/GlyA^+^ erythroid subset—suppress T-cell effector function via arginase/ROS-dependent mechanisms and checkpoint ligands, demonstrating a direct immunoregulatory role for erythroid lineage cells [13]. In patients, higher baseline CEC frequencies are associated with weaker anti-tumor responses and poorer outcomes on anti-PD-L1 therapy [80]. Tumor-conditioned erythroid progenitors can transdifferentiate into EDMCs that express ARG1/ARG2 and PD-L1 and inhibit CD8^+^ T-cells; in vivo depletion or functional blockade restores T-cell responses and partially rescues PD-1/PD-L1 sensitivity [14,30,80]. Multiple upstream drivers expand these populations: tumor-derived GM-CSF promotes systemic conversion of erythroid precursors into EDMCs [14]; inflammatory cytokines (TNFα, IL-1β) sustain stress erythropoiesis [86]; and chemotherapy-induced marrow stress enriches MEPs, enlarging the erythroid progenitor pool [87]. Consistent with this, the presence of EDMCs correlates with inferior responses to PD-1/PD-L1 blockade [30].

Hypothesized role in metastasis: Because erythroid maturation is concentrated in sinusoid-proximal, CXCL12-rich perivascular territories that overlap LepR^+^ stromal hubs commonly exploited by DTCs, CEC/EDMC programs could dampen local T-cell surveillance and contribute to checkpoint resistance within bone marrow lesions [2,13]. However, the physical co-localization of immunosuppressive erythroid subsets with DTCs in human bone metastases has not yet been demonstrated by spatially resolved mapping [2,88]. Existing multi-omics atlases of human marrow define these sinusoidal erythroid territories and provide the technical framework to test this directly in bone-metastasis tissue, but current datasets still lack tumor–erythroid co-registration [2,13,88].

### 4.4. Spatial Maps and Clinical Correlates

State-of-the-art marrow atlases map hematopoiesis across endosteal and perivascular territories and with aging, but they profile non-malignant marrow and do not co-localize DTCs with immunosuppressive erythroid subsets—so malignant niches remain unresolved [87]. Bone-metastasis studies are mostly single-cell RNA-seq without spatial co-registration; recent work in bone lesions delineates stromal/immune remodeling yet still lacks spatially resolved DTC–erythroid proximity in human tissue [30]. To date, there is no publicly available single-cell spatial transcriptomic atlas of human bone metastasis that simultaneously maps tumor, stromal, and erythroid compartments [89].

Clinical workflows mirror this gap. A retrospective study relying on aspirate/biopsy and routine histology established diagnosis and outcomes but could not prospectively assess dynamic erythroid subsets (e.g., CD71^+^ CECs or EDMCs) within lesions or their modulation by therapy [90]. Blood-based data already suggest relevance: in virus-associated solid tumors, higher CEC levels correlate with impaired anti-tumor immunity and predict inferior responses to PD-1/PD-L1 blockade [90]. Notably, the Cancer Cell study on tumor-induced EDMCs stated that the spatial proximity of EDMCs to DTCs in marrow remains unknown; although erythroid-derived suppressor cells are functionally immunosuppressive in animal models, bone metastasis-specific validation in human bone biopsy tissue is lacking [14]. A separate retrospective series likewise highlighted that routine histology precludes evaluating dynamic erythroid-lineage changes during therapy [91].

Together, these points support generating bone metastasis-specific single-cell spatial atlases that integrate tumor cells, LEPR^+^/CXCL12^+^ stromal niches, and erythroid programs (CECs/EDMC-like states). Such datasets would (i) test whether immunosuppressive erythroid cells preferentially congregate near DTCs in human lesions and (ii) link circulating CEC readouts to on-lesion erythroid immunoregulation to guide niche-targeted combinations.

### 4.5. Erythroid-Axis Interventions: EPO/EPOR, CEC Depletion, and Arginine Metabolism

In several mouse tumor models, pharmacologic arginase inhibition (e.g., CB-1158/INCB001158) enhanced intratumoral CD8^+^ T-cell infiltration and restored T-cell proliferation in vitro, making tumors more sensitive to anti-PD-1 treatment. Human CD71^+^ immature erythroid cells express PD-L1, which, in ex vivo co-culture assays, directly suppresses T-cell proliferation, indicating a targetable checkpoint function of erythroid-lineage cells [80]. In preclinical models, tumor-derived EPO and dysregulated EPO/EPOR signaling can influence the immune microenvironment. In mouse models, immunological infiltration and tumor phenotype are changed when EPO signaling is genetically or pharmacologically perturbed, suggesting that EPO signaling is a programmable axis [92]. In preclinical research, anti-PD-1/PD-L1 drugs and arginase inhibition (e.g., CB-1158) increased arginine availability, which restored T-cell activation and reduced tumor development in a synergistic manner [93].

## 5. Perivascular Stromal Subsets: Beyond Osteoblasts and Endothelial Cells

### 5.1. Leptin Receptor-Positive (LepR^+^) Stromal Cells: CXCL12 Reservoirs Supporting HSCs

In mice, LepR^+^ bone-marrow stromal cells are perivascular mesenchymal cells that supply the predominant SCF and CXCL12 required for hematopoietic support [23,94,95]. In humans, CXCL12-high LEPR^+^ CAR-like stromal cells represent a conserved counterpart that reinforces a perisinusoidal, chemokine-rich niche [22]. Importantly, LepR^+^ stroma secretes high CXCL12 that engages CXCR4 on tumor cells, mediating homing and retention in bone; blocking CXCL12–CXCR4 in vivo reduces marrow metastasis in mouse breast and prostate cancer models [31,32,33,96].

### 5.2. Other Perivascular Stromal Subsets

Besides LepR^+^ cells, other marrow niches include NG2^+^ periarteriolar cells that enrich quiescent HSCs near arterioles, CXCL12-abundant reticular (CAR) cells around sinusoids, and perivascular MSC-like subsets such as human CD146^+^ stromal cells [2,22,96,97,98]. These subsets support hematopoiesis, but direct roles in metastasis are less clear; in practice, in mouse models, DTCs appear to preferentially exploit CXCL12-rich sinusoidal niches (likely CAR/LepR^+^ stroma) rather than tightly periarteriolar NG2^+^ territories [32,99]. Given their location, CAR and LepR^+^ cells likely govern CXCR4-dependent tumor homing, whereas the contribution of arteriolar NG2^+^ niches to bone metastasis remains uncertain [2,22,96,100].

### 5.3. Roles in Metastasis: DTC Anchoring, Immune Modulation, CXCL12–CXCR4-Mediated Homing

In mouse xenograft models, DTCs can directly target and occupy the HSC niche in bone marrow, where they compete with HSCs for endosteal space; experimentally expanding niche size increases metastasis, whereas compromising the niche reduces dissemination, and DTCs can be mobilized from the niche using HSC mobilization protocols [101]. Within this niche context, osteoblast- and endothelium-derived annexin II (Anxa2) facilitates tumor adhesion and homing, and engagement of the Anxa2 axis induces expression of GAS6 receptors (AXL, Sky, Mer) on prostate cancer cells; osteoblast-produced GAS6 then inhibits proliferation, protects against chemotherapy-induced apoptosis, and shifts cell-cycle distribution toward quiescence consistent with a dormancy-supporting anchor in marrow [96].

Chemokine signaling via the CXCL12–CXCR4 axis provides a core homing and engraftment mechanism for tumor cells in bone-implicated metastasis. Reports show CXCL12 (SDF-1) is produced by marrow stromal elements and widespread CXCR4 expression on tumor cells; this axis is repeatedly implicated in tumor progression and distant metastasis, including marrow settings [100]. Neutralizing CXCL12/CXCR4 interactions in vivo significantly reduces metastatic seeding in mouse classic breast cancer models [102].

Inflammatory and Tumor-Derived Remodeling of the LepR^+^ Niche. The retention of DTCs within the bone marrow is heavily reliant on the CXCL12–CXCR4 axis, which anchors tumor cells to the perivascular LepR^+^ niche and enforces a state of cellular quiescence. However, this “chemokine trap” is not static; it is actively degraded by inflammatory signals that remodel the niche to favor metastatic outgrowth. The most potent modulator of this axis is Granulocyte-Colony Stimulating Factor (G-CSF). While often used clinically to mobilize hematopoietic stem cells, G-CSF also acts as a pro-metastatic signal when secreted by tumors or induced by chemotherapy. Mechanistically, G-CSF does not act directly on stromal cells; rather, it stimulates bone marrow monocytes and neutrophils to release proteolytic enzymes (e.g., neutrophil elastase, cathepsin G) and soluble mediators that act on LepR^+^ cells. This signaling cascade results in the profound transcriptional suppression of Cxcl12 and Kitl (SCF) in LepR^+^ stromal cells [103]. The collapse of the CXCL12 gradient releases CXCR4^+^ DTCs from their quiescent, perivascular anchors, triggering their transition from dormancy to active proliferation.

Beyond G-CSF, the inflammatory cytokines IL-1β and TNF-α, which are often elevated in the aged or tumor-bearing marrow (“inflamm-aging”), have been shown to specifically downregulate Cxcl12 expression in perivascular stromal cells [104]. This downregulation creates a “leaky” niche that fails to retain dormant cells. Furthermore, tumor-derived TGF-β creates a feed-forward loop during osteolytic bone resorption. While TGF-β can enforce dormancy via direct signaling to tumor cells, high levels of TGF-β in the matrix have been shown to suppress Cxcl12 production in mesenchymal stromal cells [105], potentially destabilizing the niche. This “two-hit” model loss of the CXCL12 retention signal combined with an inflammatory proliferative drive provides a mechanistic basis for metastatic reactivation.

More broadly, marrow-focused work describes how hypoxia-inducible CXCL12 recruits CXCR4^+^ bone-marrow–derived cells, supports tumor cell adhesion to endothelium and transendothelial migration, and promotes motility via MMPs/integrins, processes integral to vascular entry/exit and perivascular niche engagement [100].

Stromal immune modulation further promotes metastatic competence. In mouse models, bone marrow-derived mesenchymal stem/stromal cells (MSCs) can enhance metastasis of breast cancer cells through a CCL5–CCR5 paracrine loop; genetic or antibody disruption of CCR5/CCL5 abrogates MSC-induced metastasis and increases extravasation deficits [106]. Additionally, MSC metabolic programming via lysosomal acid lipase (LAL) regulates secretion of IL-6, MCP-1, and IL-10; In mice, LAL deficiency reduces these cytokines, impairs MSC-stimulated tumor growth and metastasis, increases CD8^+^ T cells, and decreases tumor-promoting Ly6G^+^CD11b^+^ myeloid-derived suppressor cells, indicating that intact MSC signaling favors tumor progression and metastatic spread through immunosuppressive circuits [107].

Upstream oncogenic programs can potentiate chemokine-guided dissemination. In cell culture and mouse models of breast cancer, HER2 overexpression increases CXCR4 expression (by enhancing synthesis and inhibiting ligand-induced degradation), and CXCR4 is required for HER2-driven lung metastasis; high CXCR4 correlates with poorer overall survival [108]. These data, taken together, show that DTC anchoring (Anxa2–GAS6), immune modulation (MSC-derived CCL5/CCR5 and cytokines), and CXCL12–CXCR4–mediated homing cooperate to enable niche engagement during metastasis [96,100,101,102,106,107,108,109].

### 5.4. Heterogeneity Gaps: Functional Specialization Along Arteriolar vs. Sinusoidal Niches Not Fully Mapped in Bone Metastases

Arteriolar and sinusoidal niches are functionally distinct in healthy mouse marrow: small-caliber endosteal arterioles ensheathed by NG2^+^ perivascular cells maintain quiescent HSCs, while sinusoidal vessels with LepR^+^, CXCL12-rich stromal cells support more proliferative and mobilizable HSC pools [110,111].

In bone metastasis, however, this arteriolar–sinusoidal spectrum has not been systematically mapped. Existing data show dormant breast cancer micrometastases localized near CXCL12^+^ perisinusoidal vessels and reliant on CXCR4/E-selectin interactions in mice, while osteolytic lesions display expanded αSMA^+^ arterial networks and arterial/axon-guidance programs in the stromal compartment [99,112]. These observations come from separate models and disease contexts, leaving a clear heterogeneity gap: we still lack an integrated spatial and functional map of how DTCs differentially exploit arteriolar versus sinusoidal niches during dormancy and outgrowth in bone.

This gap can be addressed by adapting tools already validated in hematopoietic biology and metastasis: 3D whole-mount imaging in mice with computational spatial modeling of vessel subtypes, intravital tracking of DTCs, and targeted perturbation of CXCL12–CXCR4/E-selectin and arterial guidance pathways in bone metastasis models [99,110,111,112]. Systematically applying these approaches would generate unified arteriolar vs. sinusoidal niche maps and clarify how hematopoietic niche hijacking is partitioned across vascular subsets in bone metastases.

### 5.5. Potential Therapeutic Entry Points: CXCL12–CXCR4 Axis Inhibition (Plerixafor), Niche Disruption Strategies

Targeting the CXCL12–CXCR4 axis has shown disease-modifying effects in bone-tropic models of prostate cancer (PCa). In murine intracardiac and intratibial PCa models, pharmacologic CXCR4 blockade with plerixafor reduced the incidence of radiographic bone lesions, decreased osteolysis (lower CTX-I and TRAP-5b), and improved overall survival compared with vehicle controls [31]. In the same study, CXCR4 peptide antagonist (CTCE-9908) similarly reduced bone lysis and systemic bone-resorption markers, with trends towards lower lesion incidence, supporting on-target anti-metastatic activity in bone [31].

Preclinical work in other tumor contexts aligns with these findings. In osteosarcoma and melanoma experimental metastasis models, CTCE-9908 reduced gross pulmonary metastases by ~50–56% and, when dosed before tumor-cell injection, achieved up to an ~81% reduction in lung nodules, indicating effective disruption of CXCL12–CXCR4–mediated seeding and early outgrowth [113]. “Mobilize-to-sensitize” niche-disruption strategies further support CXCR4 inhibition as a therapeutic entry point. In breast cancer bone-marrow micrometastasis, SDF-1 (CXCL12) acts as a molecular tether within sinusoidal, E-selectin^+^ pro-dormancy niches; the E-selectin inhibitor GMI-1271 reduced metastatic entry into bone, while the CXCR4 antagonist AMD-3100 (plerixafor) mobilized established marrow disease out of these niches, nominating a combinable approach to purge BM reservoirs [32]. Combining CXCR4 blockade with chemotherapy can enhance anti-tumor activity in bone. A dual E-selectin/CXCR4 antagonist (GMI-1359) decreased bone-marrow colonization and intra-osseous tumor growth in PCa models and sensitized to docetaxel, with concomitant reductions in osteolysis and serum resorption markers (mTRAP, CTX) compared with single-pathway antagonists (CTCE-9908 or GMI-1271) [114]. In a complementary intratibial PCa model, the selective CXCR4 inhibitor balixafortide added to docetaxel produced greater anti-tumor effects than either agent alone, with supportive biomarker changes providing a rationale to evaluate CXCR4 inhibition as a docetaxel adjuvant in bone-metastatic PCa [33].

Finally, ligand-level disruption of the axis is clinically feasible. In relapsed/refractory multiple myeloma, the CXCL12-neutralizing Spiegelmer olaptesed pegol (NOX-A12) safely mobilized malignant plasma cells for ≥72 h and augmented bortezomib–dexamethasone activity (overall response rate 68%), consistent with the concept that CXCL12 blockade can detach tumor cells from supportive marrow niches while enhancing systemic therapy [115].

## 6. Crosstalk Between Niche Components in Metastasis

### 6.1. Nterplay Driving Bone Metastasis: MKs, Erythroid Cells, Stroma, and Bone Cells

(A) MK–stromal–osteoblast crosstalk

**Figure 1:**
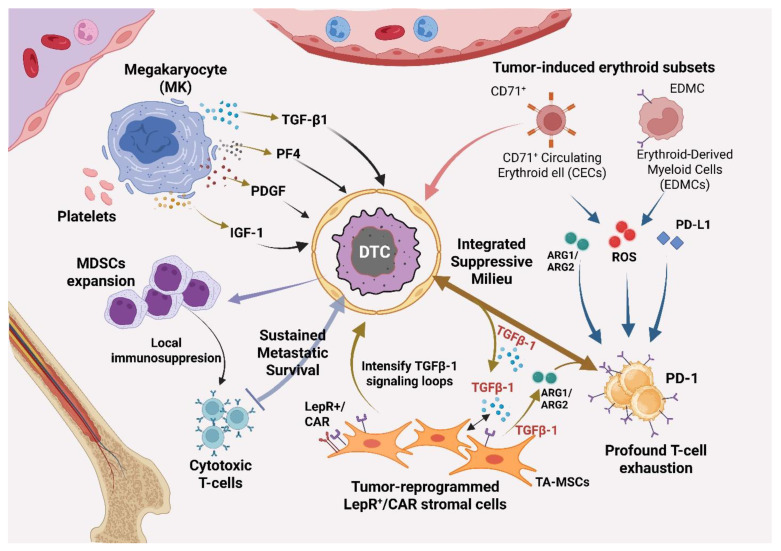
Integrated immune-suppressive niche in bone metastasis. This schematic summarizes how DTCs co-opt hematopoietic niches. High-ploidy megakaryocytes (MKs) and platelets secrete TGF-β1 (transforming growth factor β1), PF4 (CXCL4), PDGF, and IGF-1, which expand myeloid-derived suppressor cells (MDSCs) and intensify local TGF-β loops. Concurrently, tumor-induced CD71^+^ immature erythroid cells, including circulating erythroid cells (CECs) and EDMCs, accumulate and express immunoregulatory mediators (arginases ARG1/ARG2, reactive oxygen species, checkpoint ligand PD-L1) that blunt CD8^+^ T-cell effector function. A protective stromal niche of LepR^+^ CXCL12-abundant reticular (CAR) cells anchor the DTCs via CXCL12 gradients. Together, these converging signals produce PD-1/PD-L1 checkpoint engagement and profound T-cell exhaustion, sustaining metastatic survival. Abbreviations: CEC, CD71^+^ immature erythroid cell; EDMC, erythroid-derived myeloid cell; LepR, leptin receptor; CAR, CXCL12-abundant reticular; MDSC, myeloid-derived suppressor cell; PF4, platelet factor 4; PD-1, programmed cell death protein 1; TGF-β1, transforming growth factor β1; TA-MSC, tumor-associated mesenchymal stromal cell. Created in BioRender. Mohammad, K. (2026) https://BioRender.com/yj8rdob.

MK α-granules store TGF-β1, PDGF, and IGF-1, enabling rapid paracrine signaling upon activation [116]. Functionally, MKs are a source of CXCL4 (PF4) that enforces HSC quiescence in neighboring cells, illustrating direct MK-to-niche control [117]. At the bone interface, MKs promote osteoblast proliferation and constrain osteoclastogenesis, shifting bone remodeling toward formation and stabilizing perivascular/endosteal microenvironments: MKs enhance osteoblast proliferation [72] and inhibit osteoclast formation in co-culture and in vivo [70]. High-ploidy, large-cytoplasmic MKs further act as niche regulators in marrow [19], while a specialized MK-surrounding ECM “cage” physically anchors MKs near sinusoidal outlets, an architecture that optimizes proplatelet release and positions MK-derived cues to influence adjacent stromal/osteolineage cells [37] (Figure 1).

(B) MK–erythroid interactions and shared suppressive outputs

Platelets and MKs provide an immunoregulatory layer via TGF-β1, which reprograms myeloid suppressors and shapes immune tone [42]. In parallel, immature erythroid populations exert potent T-cell suppression. CECs suppress T-cell effector functions in humans via contact-dependent and metabolic mechanisms (e.g., ARG1/ARG2, ROS, checkpoint ligands) [80]. Tumors can reprogram erythroid progenitors into EDMCs that express PD-L1 and ARG1/ARG2, inhibiting T-cell activity and curtailing anti-PD-1/PD-L1 responses [14]. Together, MK/platelet TGF-β1 and erythroid PD-L1/ARG1 establish a convergent suppressive network within marrow and metastasis-adjacent niches [14,42,80].

(C) Immune-suppression convergence (TGF-β, PD-L1, ARG) and therapeutic levers

Three recurrent axes connect these compartments: (i) TGF-β from MKs/platelets tunes stromal and immune states [42]; (ii) PD-L1 on EDMC-like cells dampens T-cell activation; (iii) arginine depletion via ARG1/ARG2 disables T-cell metabolism [14]. Genetic and pharmacologic work validates arginase as a tractable node—blocking ARG activity restores antitumor T-cell function in preclinical systems and shows early clinical promise [118]. These convergences motivate rational combinations pairing niche-targeted TGF-β pathway modulators with arginase inhibition to relieve layered suppression [91].

(D) Expanded erythroid role: niche residency, triggers, and metabolic competition

Erythropoiesis in adult marrow is organized as sinusoid-proximal “islands” supported by perivascular stroma [63]. Human data show CD71^+^ CECs as quantifiable, immunosuppressive cells (flow: CD71^+^/GlyA^+^) that correlate with impaired antitumor immunity in patients [80]. Tumors expand and reprogram erythroid trajectories: single-cell lineage and functional experiments demonstrate tumor-driven conversion of erythroid progenitors into EDMCs with PD-L1/ARG programs that blunt checkpoint efficacy [14]. These expansions heighten local nutrient demand by erythroid lineage cells (which are intrinsically high-CD71/iron-avid), plausibly reshaping metabolic competition in sinusoidal niches; in parallel, tumor cytokine cues (e.g., TGF-β–rich platelet/MK milieu) provide a permissive context for erythroid-programmed immunosuppression [14,42,80]. Collectively, this positions CECs/EDMC-like signatures as candidate liquid biomarkers for monitoring erythroid-driven immune evasion and stratifying immunotherapy response [14,80].

(E) Niche Convergence and Therapeutic Levers

Hematopoietic, stromal, and bone-derived cells continuously exchange molecular signals within the bone marrow niche, which operates as an integrated ecosystem. Megakaryocytes demonstrate marked transcriptional heterogeneity, according to single-cell data, but defining their function requires understanding their spatial context. Solid tumors include platelet-derived microparticles (PMPs), which can transmit proteins and RNAs to tumor cells and rewire recipient cell transcriptional programs in vivo [119]. Proteomic and secretome profiling indicate that megakaryocytes play a role in stromal reprogramming; they store TGF-β1, PDGF, and IGF1 in α-granules and release these mediators upon activation [120]. Single-cell datasets identify megakaryocytes as a major source of TGFB1 transcripts in marrow, and MK ablation reduces bone-marrow bioactive TGF-β protein and downstream pSMAD2/3 signaling in neighboring cells. In addition to megakaryocytes’ function in niche remodeling, another hematopoietic lineage contributes significantly to the barrier’s immunosuppressive properties: CECs and those that infiltrate tumors suppress T-cell proliferation and IFN-γ production via reactive oxygen species and PD-L1 expression [80]. Pharmacologic arginase blockade or genetic interference reverses this suppression and restores antitumor T-cell function in preclinical models, demonstrating the immunomodulatory relevance of erythroid ARG1 [13,83,84]. Importantly, osteoclast-driven bone resorption liberates matrix-sequestered TGF-β, which amplifies niche immunosuppression and tumor survival programs in a feed-forward loop [121].

### 6.2. Hypothesis: Synergistic “Suppression Circuits” (TGF-β + PD-L1/Arg-1 + CXCL12)

In mouse models of ITP, platelet/MK-derived TGF-β1 produced after activation promotes immunological suppression by inducing MDSCs expansion and functional reprogramming through canonical SMAD2/3 signaling [42]. In both co-culture and ex vivo settings, CECs and marrow erythroid progenitors in cancer patients exhibit immunoregulatory markers (ARG1, PD-L1) and effectively inhibit CD8^+^ T-cell proliferation and IFN-γ production [80]. To validate its function in immune evasion, this cooperative system requires combinatorial treatment testing. In preclinical investigations, TGF-β neutralization can alleviate immune suppression without causing widespread systemic damage, as seen by the reactivation of CD8^+^ T-cells and the reduction in tumor burden in mice models when TGF-β signaling is blocked (selective methods like anti-GARP:TGF-β1 antibodies) [47]. Arginase is a druggable node in immune suppression circuits as pharmacologic inhibition of the enzyme (such as that of the CB-1158/NB-001 family in preclinical investigations) reverses arginase-dependent suppression of T-cells and can enhance anti-tumor immunity [122]. In animal models, anti-GARP:TGF-β1 antibodies increase T-cell infiltration and function in conjunction with immune checkpoint inhibition (anti-PD-1), suggesting that TGF-β neutralization in combination with other immune modulators improves efficacy [48].

## 7. Therapeutic and Translational Implications of Niche–Immune Crosstalk

### 7.1. Biomarkers of Bone Involvement

In patients with virus-associated solid tumors, circulating CECs are markedly increased, and their baseline frequency is correlated with a poor response to anti-PD-L1 therapy [80]. A 15-gene expression pattern from initial breast carcinomas may represent stromal and tumor cell gene programs linked to skeletal colonization and predicted eventual bone metastases with >80% sensitivity [123]. A stroma-derived metastasis signature (SDMS) of ~93 genes in prostate cancer was independently prognostic of metastasis and highlights that stromal gene expression may function as a liquid or tissue biomarker of metastatic risk [124]. In multiple myeloma, an increase in CD71^+^ erythroid precursors with high ARG2 expression was linked to poorer survival and disease progression, indicating the possibility of prognostic biomarkers in malignancies affecting the marrow [125]. The presence of niche-specific biomarker signatures was supported by single-cell analysis of bone-metastatic versus benign marrow in clear-cell renal-cell carcinoma, which showed differential stromal and immunological remodeling in the bone involvement microenvironment [30].

### 7.2. Megakaryocyte-Driven Immunoregulation and Therapeutic Targeting

In preclinical settings, platelet-derived TGF-β1 links MK/platelet activation to systemic immune suppression by promoting the growth and functional reprogramming of MDSCs through canonical TGF-β/SMAD signaling [42]. In mouse models, platelet GPVI has been identified as a targetable platelet receptor whose blockade decreases the growth of established metastases while maintaining significant hemostatic function. This suggests that platelet-focused inhibition can impede metastatic progression without posing an excessive risk of bleeding [73].

### 7.3. Targeting Niche Adhesion: Lessons from the FORTRESS Trial and Dual Inhibition

The strategy of “mobilize-to-sensitize”, detaching tumor cells from their protective niche to render them susceptible to chemotherapy, has reached critical junctures in clinical development. Balixafortide, a potent selective CXCR4 antagonist, showed promise in early-phase studies but failed to meet its co-primary endpoints of objective response rate (ORR) and progression-free survival (PFS) in the Phase 3 FORTRESS trial (NCT03786094) for HER2-negative metastatic breast cancer [126]. This failure highlights the complexity of targeting established, bulky metastases where tumor cells may no longer be strictly dependent on the CXCR4–CXCL12 interaction for survival, or where systemic CXCR4 blockade might disrupt anti-tumor immune trafficking.

Consequently, the focus has shifted toward dual-targeting and earlier intervention. GMI-1359, a dual antagonist of both E-selectin and CXCR4, targets both the initial vascular entry [114]. Although a Phase 1b study in bone-metastatic breast cancer (NCT04197999) was terminated early due to enrollment challenges, it successfully demonstrated the mobilization of CD34^+^ cells and pharmacodynamic target engagement [127], validating the mechanism in humans. Future success in this class likely requires application in the minimal residual disease (MRD) setting rather than advanced metastatic disease.

### 7.4. Dismantling the Immunosuppressive Barrier: Platelet/Treg GARP–TGF-β and Erythroid Arginase Checkpoints

#### 7.4.1. Selective Blockade of Platelet/Treg-Activated TGF-β (anti-GARP:TGF-β1; Livmoniplimab)

Targeting the specific molecular barriers erected by megakaryocytes and erythroid cells offers a more precise immunotherapy approach. Traditional TGF-β inhibitors have been hampered by systemic toxicity, but Livmoniplimab (ABBV-151), a monoclonal antibody targeting the GARP:TGF-β1 complex, offers a solution. By specifically blocking the release of active TGF-β1 from the surface of platelets and regulatory T cells (Tregs) without inhibiting latent TGF-β elsewhere, it minimizes toxicity. Recent Phase 1 data (NCT03821935) have shown that Livmoniplimab can deepen responses to PD-1 blockade in checkpoint-refractory solid tumors [50], validating the hypothesis that platelet/megakaryocyte-derived TGF-β is a reversible brake on immunity. Parallel to this, metabolic checkpoints governed by erythroid lineage cells are being targeted via Arginase inhibition. INCB001158 (Numidargistat), a small-molecule arginase inhibitor, aims to reverse the L-arginine depletion caused by ARG2-expressing erythroid cells and myeloid-derived suppressor cells. While monotherapy activity has been limited, combination trials with pembrolizumab (NCT02903914) have shown durable responses in microsatellite-stable colorectal cancer and other solid tumors [128], suggesting that anti-GARP limits local bursts of bioactive TGF-β from platelet/MK and Treg surfaces, while arginase inhibition restores the amino-acid sufficiency required for marrow-infiltrating T-cells to function in CXCL12-rich perivascular zones.

#### 7.4.2. Reversing Arginine Starvation (Arginase Inhibitors; Numidargistat/INCB001158)

**Figure 2:**
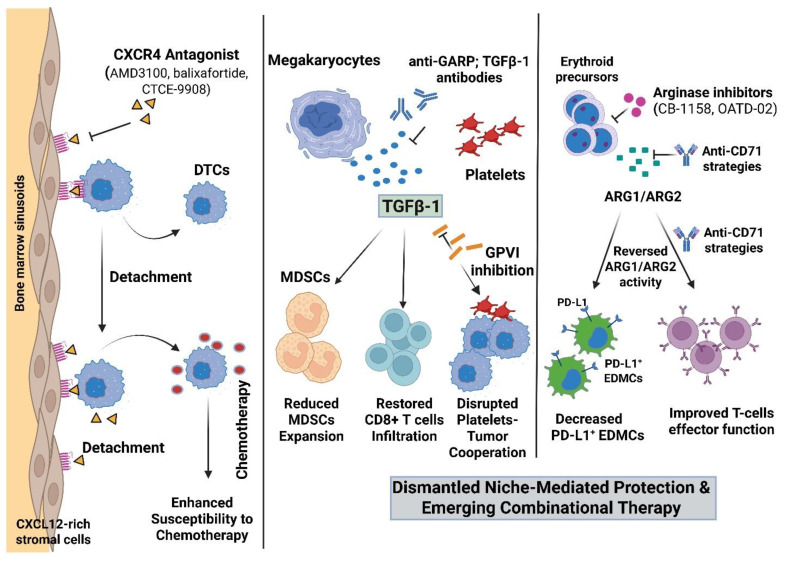
Strategies to dismantle bone marrow niche protection and sensitize metastases. (**Left**) Niche detachment: CXCR4 antagonists (e.g., AMD3100/plerixafor, balixafortide, CTCE-9908) disrupt CXCL12-mediated retention of DTCs in marrow sinusoids, mobilizing tumor cells and enhancing chemotherapy efficacy. (**Center**) Anti-platelet/MK therapy: Blocking platelet TGF-β1 activation with anti-GARP antibodies, together with GPVI inhibition, interrupts tumor–platelet crosstalk. This reduces MDSC expansion and allows CD8^+^ T-cell infiltration. (**Right**) Erythroid axis interventions: Arginase inhibitors (CB-1158, OATD-02) and anti-CD71 antibodies reverse the metabolic suppression driven by tumor-reprogrammed CD71^+^ erythroid cells. These therapies decrease immunosuppressive PD-L1^+^ EDMC numbers and improve T-cell effector function. Abbreviations: CXCL12, CXC chemokine ligand 12; DTC, disseminated tumor cell; GPVI, platelet glycoprotein VI; GARP, glycoprotein-A repetitions predominant (latent TGF-β1 receptor); MDSC, myeloid-derived suppressor cell; PD-L1, programmed death ligand 1; TGF-β1, transforming growth factor β1; ARG1/ARG2, arginase-1/2. Created in BioRender. Mohammad, K. (2026) https://BioRender.com/6cv02fi.

Metabolic and cellular mechanisms of immune evasion within the tumor microenvironment are emerging as targetable drivers of checkpoint resistance. A molecular rationale for arginase inhibition (CB-1158, OATD-02) comes from preclinical data showing restoration of intratumoral arginine, increased CD8^+^ T-cell infiltration, and synergy with PD-1 blockade [83,84,122]. Combination testing with checkpoint inhibitors is supported by first-in-human and early clinical data for arginase inhibitors (INCB001158/CB-1158), which show pharmacodynamic target engagement (increased plasma arginine) and tolerability [128]. The functional benefit of selective depletion is demonstrated by the rescue of reduced CD8^+^ T-cell proliferation and the restoration of efficient antiviral or anticancer immunity in murine models when CD71^+^ erythroid cells with anti-CD71 antibodies are depleted [84,129]. Newer dual inhibitors (like OATD-02) and next-generation compounds that target both intracellular and extracellular ARG1 are being developed, specifically to overcome resistance in hypoxic tumors and expand the range of situations where arginase blockage might restore antitumor immunity [130] (Figure 2).

### 7.5. EPO Signaling Modulation

EPO can be produced by tumor cells in various settings. In murine hepatocellular carcinoma models, conditional deletion of tumor-derived EPO decreased tumor development and increased CD8 T-cell infiltration [92]. Pharmacologic blockage or genetic deletion of EPOR reprogrammed tumor-associated macrophages (TAMs) away from an immunoregulatory phenotype, which boosted anti-tumor T-cell responses and slowed tumor growth in vivo [92]. A major safety limitation for therapeutic blocking is that systemic targeting of EPO signaling might cause anemia; animal models show that total EPO/EPOR inhibition lowers hemoglobin and erythropoiesis levels [131,132].

## 8. Future Directions and Knowledge Gaps

### 8.1. High-Priority Questions

Which niche subsets (MKs, erythroid cells, LepR^+^ stromal cells) are engaged first by DTCs, and does this sequence depend on tumor type or treatment history? Evidence that human DTCs target marrow vascular/HSC territories and that endothelial E-selectin can reprogram cancer cells toward bone fitness suggests early engagement of vascular and perivascular ensembles (LepR^+^/CAR stroma, endothelium), but precise ordering across tumor histology remains unresolved [21,22,94,101].

The temporal dynamics of niche hijacking remain unclear: dormant–reactivation cycles in bone marrow suggest that stromal and hematopoietic reprogramming can precede or follow micrometastatic seeding depending on the context, yet time-resolved human data are lacking [99].

Do metastasis-promoting cues differ by tumor lineage? Single-cell profiling of renal cell carcinoma bone lesions shows stromal/immune remodeling distinct from non-osseous sites, hinting that engagement hierarchies may be cancer-specific [30].

How does prior therapy reshape “entry points” to the niche? Chemotherapy and diet-primed platelet activation can preconfigure pre-metastatic niches, potentially changing which subsets (MKs, LepR^+^ stroma, erythroid lineages) are co-opted first [49].

Can niche-focused interventions synergize with immunotherapy to eliminate marrow DTCs? Dual E-selectin/CXCR4 antagonism and CXCR4 blockade reduce intraosseous colonization in preclinical models; arginase inhibition restores T-cell function and is tolerable in early trials; and AhR-axis perturbations are mechanistically implicated in erythroid/myeloid reprogramming, collectively suggesting rational combinations with PD-L1 blockade [57,113,114,122,128].

### 8.2. Needed Methods and Studies

Single-cell and spatial omics directly on human bone metastasis (tumor + immune + stromal + hematopoietic components) are needed to map cell–cell interactions and DTC proximity to MKs, CECs/EDMCs, and LepR^+^ stroma in situ, building on marrow spatial atlases and metastasis single-cell datasets [2,30,60].

These atlases should incorporate perivascular topology (arteriolar vs. sinusoidal) and maturation states of MKs (including high-ploidy subsets) to test whether thrombopoietic programs physically align with DTC “handoff” zones [19,60].

Genetic lineage tracing of MKs, erythroid progenitors, and LepR^+^ stromal cells in bone metastasis models is required to assign causal roles in seeding, dormancy, and reactivation. New multimodal barcoding approaches (e.g., CellTag-multi) can capture fate switches and regulatory rewiring during tumor–niche crosstalk [19,67,94].

Longitudinal human sampling that pairs blood (CTCs, CECs/EDMC-like readouts) with marrow aspirates/biopsies before and after therapy can reveal dynamic tissue remodeling, link circulating biomarkers to tissue states, and identify responders to niche-targeted combinations [56,80].

Interventional studies should prospectively evaluate whether CXCR4/E-selectin antagonists or arginase/AhR pathway inhibitors augment checkpoint therapy in patients with marrow involvement, with correlative tissue and liquid-biopsy endpoints to confirm on-target niche remodeling [57,114,122,128].

Finally, standardized analytical frameworks (ligand–receptor inference, spatial neighborhood statistics) are needed across centers to compare bone-lesion niches by tumor type and treatment exposure, enabling reproducible maps of “first responders” in niche hijacking [2,30].

## 9. Conclusions

The resistance of bone metastases to current standard treatments highlights a critical need to reframe our understanding of skeletal colonization. It is becoming increasingly clear that the bone marrow is not a passive recipient of metastasis but a highly active immunological organ whose regulatory machinery is systematically hijacked by tumor cells. This review emphasizes that the hematopoietic niche extends far beyond the classical osteoblast/osteoclast-tumor interaction. Megakaryocytes and erythroid lineage cells emerge as pivotal “co-conspirators”, providing the signals required for dormancy, immune evasion, and metabolic support that enable disseminated cells to persist despite adjuvant therapy. Specifically, the ability of megakaryocytes to enforce a quiescent, stem-like state in tumor cells via TGF-β1 mimics their physiological role in maintaining HSCs, effectively shielding cancer cells from chemotherapeutic insults. Concurrently, the co-option of erythroid progenitors to suppress local immunity reveals a sophisticated metabolic strategy where tumors utilize the body’s own stress response to blunt T-cell surveillance. The definition of these distinct stromal and hematopoietic subsets suggests that the “soil” is highly heterogeneous, with specific arteriolar and sinusoidal vascular niches likely governing the critical switch between dormancy and aggressive outgrowth.

Future clinical translation must therefore pivot toward disrupting these support systems. Therapeutic avenues that target the niche itself, such as blocking TGF-β activation on platelets via GARP inhibition, or reversing erythroid-driven immune suppression with arginase inhibitors represent a necessary shift from attacking the seed to conditioning the soil. To fully realize this potential, the field must advance toward spatially resolved mapping of human metastatic tissue to confirm that the distinct “suppression circuits” identified in murine models are active drivers of patient disease. By dismantling the niche protections that tumor cells rely upon, we may finally render bone metastases vulnerable to eradication.

## Figures and Tables

**Table 1:** Early versus late bone metastasis: dominant niche cell types and signaling programs. Early disease is enriched for marrow homing/retention and immune-evasive dormancy circuits (E-selectin, CXCL12–CXCR4, MK/platelet TGF-β, erythroid PD-L1/arginase), whereas late disease features reactivation with stromal remodeling, osteoclast-driven osteolysis, and amplification loops that support tumor expansion.

Biologic Theme	Early Bone Metastasis (Entry → Niche Capture → Dormancy/Micrometastasis)	Late Bone Metastasis (Reactivation → Lesion Expansion → Osteolysis/“Vicious Cycle”)
Dominant tumor state	DTC homing, perivascular/endosteal anchoring, quiescence/drug tolerance	DTC proliferation, EMT/stemness programs, macrometastatic growth
Key anatomic “address”	Perivascular sinusoidal and HSC-like niches; CXCL12-rich stromal territories	Remodeled metastatic stroma and bone-destructive lesion microenvironments
Endothelium/adhesion	E-selectin–mediated marrow entry; endothelial capture and early lodging	Sustained vascular remodeling/angiocrine support (context-dependent)
Core chemokine axis	CXCL12–CXCR4: retention/“chemokine trap” supporting DTC persistence	Inflammation/proteases suppress CXCL12, loosening retention and enabling outgrowth (reactivation context)
Perivascular stroma (LepR^+^/CAR)	LepR^+^/CXCL12-abundant stroma: DTC anchoring, HSC-like sanctuary signaling	Emergence of tumor-associated MSC-like programs that favor osteoclast-supportive cues and immune suppression
Osteoblast/endosteal dormancy signals	Anxa2–GAS6–AXL/Sky/Mer axis promotes dormancy-like quiescence and survival	Loss/override of dormancy constraints; tumor and stromal cues become growth-permissive
Megakaryocytes/platelets (MK axis)	Platelet/MK shielding of tumor cells; TGF-β1–SMAD2/3 can support quiescence and immune suppression; PF4/CXCL4-like quiescence tone (conceptual parallel to HSC control)	Platelet/MK TGF-β becomes pro-metastatic: EMT/stemness/angiogenesis; platelet–tumor aggregates promote dissemination and lesion support
Erythroid lineage (CEC/EDMC axis)	Expansion of CD71^+^ erythroid cells; ARG1/ARG2-mediated arginine depletion, ROS, PD-L1/PD-1 checkpoint engagement → local T-cell suppression in marrow niches	Persistent erythroid-derived immune suppression can reinforce checkpoint resistance in established lesions (especially if maintained by inflammation/therapy stress)
Myeloid suppression	MDSC functional enhancement downstream of TGF-β signaling; early immune evasion in marrow	Myeloid-dominant immunosuppressive milieu supports tumor expansion and therapy resistance
Inflammatory “reactivation” triggers	Typically lower; dormancy favored by intact retention cues	G-CSF/IL-1β/TNF-α (and related remodeling) can destabilize CXCL12 retention; inflammatory remodeling enables proliferation
Bone remodeling (osteoclast/osteoblast)	Often subclinical/limited; niche biology dominates	Osteoclast activation → osteolysis → growth factor release (classical late vicious cycle emphasis)
Signature pathways to highlight in figures/tables	E-selectin; CXCL12–CXCR4; TGF-β1–SMAD (MK/platelet); PD-1/PD-L1; ARG1/ARG2; GAS6–AXL	RANKL/RANK/OPG imbalance (vicious cycle framing); inflammatory suppression of CXCL12; high TGF-β activity; sustained checkpoint + metabolic suppression

## Data Availability

No new data were created or analyzed in this study.

## Abbreviations

AhR	Aryl hydrocarbon Receptor
ANGPTL2	Angiopoietin-like 2
ARG1/ARG2	Arginase-1/Arginase-2
CAR (cells)	CXCL12-Abundant Reticular cells
CEC(s)	CD71^+^ Erythroid Cells (immature erythroid cells)
CTC(s)	Circulating Tumor Cells
CTX/CTX-I	C-terminal telopeptide of type I collagen (bone resorption marker)
CXCL12 (SDF-1)	C-X-C motif chemokine ligand 12 (Stromal cell–Derived Factor-1)
CXCR4	C-X-C chemokine Receptor 4
DTC(s)	Disseminated Tumor Cells
EDMC(s)	Erythroid-Derived Myeloid Cells
EMT	Epithelial-to-Mesenchymal Transition
EPO/EPOR	Erythropoietin/Erythropoietin Receptor
GARP	Glycoprotein-A Repetitions Predominant (latent TGF-β1 docking receptor)
G-CSF	Granulocyte Colony-Stimulating Factor
GPVI	Glycoprotein VI (platelet collagen receptor)
HSC(s)	Hematopoietic Stem Cells
IFN-γ	Interferon-gamma
IL-6/IL-10	Interleukin-6/Interleukin-10
LAL	Lysosomal Acid Lipase
LepR/LepR^+^	Leptin Receptor/cells positive for leptin receptor (gene: LEPR)
LCM(s)	Large-Cytoplasmic (high-ploidy) Megakaryocytes
MCP-1 (CCL2)	Monocyte Chemoattractant Protein-1
MDSC(s)	Myeloid-Derived Suppressor Cells
MEP(s)	Megakaryocyte–Erythroid Progenitors
MK(s)	Megakaryocytes
MMP(s)	Matrix Metalloproteinases
M-CSF	Macrophage Colony-Stimulating Factor (CSF-1)
MSC(s)	Mesenchymal Stem/Stromal Cells
CNOX-A12	Olaptesed pegol (CXCL12-neutralizing Spiegelmer)
PD-1/PD-L1/PD-L2	Programmed cell death-1/ligands 1 & 2
PF4 (CXCL4)	Platelet Factor-4 (chemokine CXCL4)
pSMAD2/3	Phosphorylated SMAD2/3 (TGF-β pathway effectors)
SCF (KITL)	Stem Cell Factor (KIT ligand)
TGF-β	Transforming Growth Factor-beta
TPO	Thrombopoietin
TRAP-5b	Tartrate-Resistant Acid Phosphatase-5b (bone resorption marker
αSMA	alpha–smooth muscle actin
AMD3100	Plerixafor (CXCR4 antagonist)
Anxa2	Annexin II
AXL/Sky/Mer	TAM-family receptor tyrosine kinases
BM/BMSC(s)	Bone Marrow/Bone-Marrow Mesenchymal Stromal Cells
E-selectin	Endothelial selectin (CD62E)
GAS6	Growth Arrest–Specific 6
HER2	Human Epidermal Growth Factor Receptor 2
PCa	Prostate Cancer
PMP(s)	Platelet-Derived Microparticles
ROS	Reactive Oxygen Species
SDMS	Stroma-Derived Metastasis Signature
TNF-α/IL-1β	Tumor Necrosis Factor-alpha/Interleukin-1 beta
